# Expression of Concern: Up-regulated microRNA-500a enhances hepatocarcinoma metastasis by repressing PTEN expression

**DOI:** 10.1042/BSR-2017-0837_EOC

**Published:** 2023-02-09

**Keywords:** AKT, Hepatocarcinoma, Metastasis, Mir-500a, PTEN

The Editorial Office has been made aware of potential issues surrounding the scientific validity of this paper, hence has issued an expression of concern to notify readers whilst the Editorial Office investigates. The Figure 3C Inhibitor NC panel seems to appear in other papers by unrelated authors, specifically as the Figure 2D Si-Tcf3#3 image from He et al. 2019 (doi: 10.1042/bsr20180369) and as the 143B/sh-VAV3+inhibitor panel of Figure 6E from Xiao et al. 2020 (doi: 10.18632/aging.103762).

